# Supplementary material for: Shared Treg dysregulation underlies psoriasis vulgaris and atopic dermatitis through the PHF19-PRC2 epigenetic axis
Source: Front Immunol. 2026 Aug 20;17:1932788. doi: 10.3389/fimmu.2026.1932788 (PMC13538097; doi:10.3389/fimmu.2026.1932788)
Supplement: Supplementary file 3 [file DataSheet3.zip › Additional files/Additional file 4/31.NTPCR.leaveoneout.pdf]

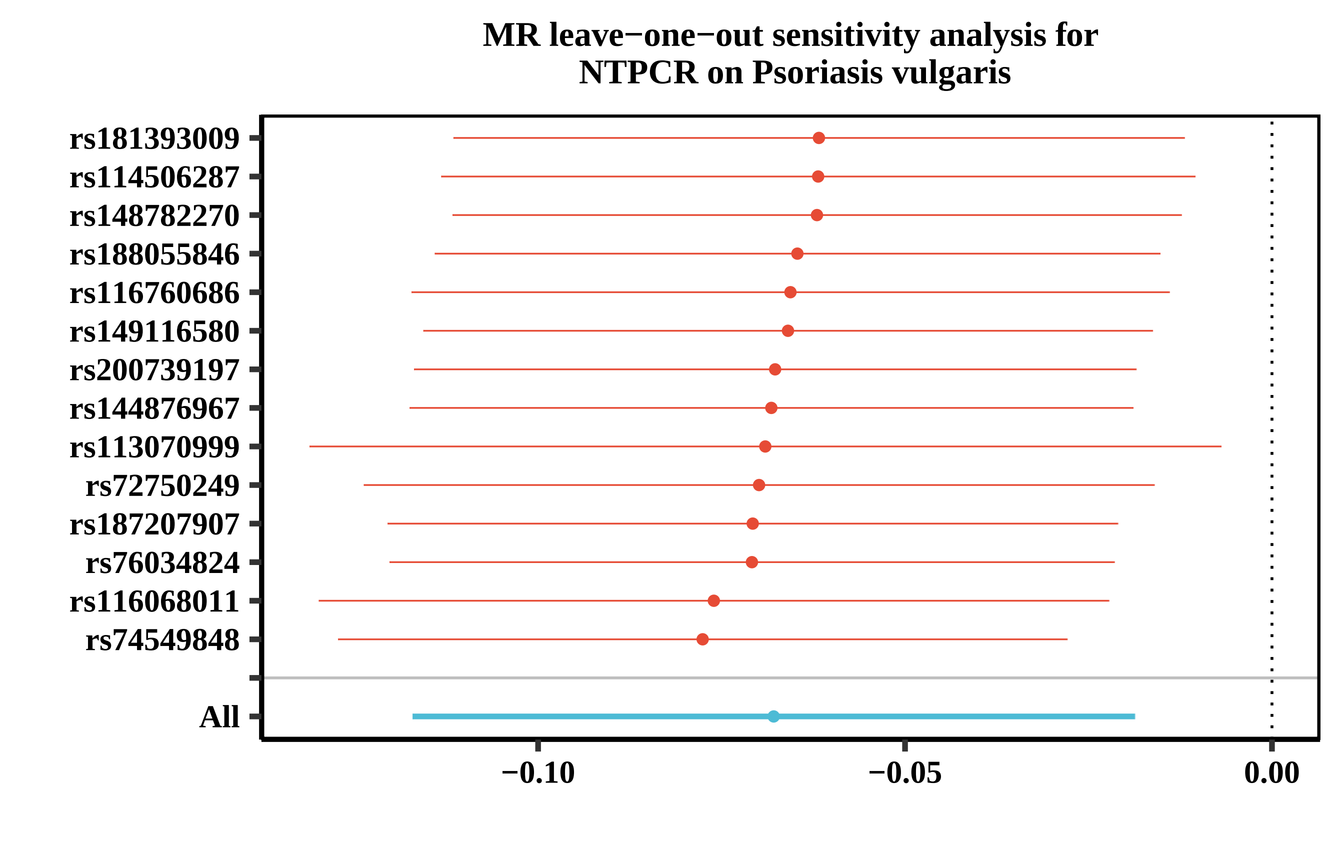
MR leave−one−out sensitivity analysis for
NTPCR on Psoriasis vulgaris
rs181393009
rs114506287
rs148782270
rs188055846
rs116760686
rs149116580
rs200739197
rs144876967
rs113070999
rs72750249
rs187207907
rs76034824
rs116068011
rs74549848
All
−0.10
−0.05
0.00
